# Diagnosing renal involvement in connective tissue disease: interpretation of anti-nuclear autoantibody tests

**DOI:** 10.1093/ndt/gfz128

**Published:** 2019-07-11

**Authors:** Ailish Nimmo, Charu Chopra, Robert W Hunter

**Affiliations:** 1 Department of Renal Medicine, Royal Infirmary of Edinburgh, Edinburgh, UK; 2 Department of Clinical Immunology, Royal Infirmary of Edinburgh, Edinburgh, UK; 3 Department of Cardiovascular Science, Queen’s Medical Research Institute, University of Edinburgh, Edinburgh, UK

## INTRODUCTION

Connective tissue diseases (CTDs) are characterized by immune system dysregulation, circulating autoantibodies and multisystem disease. Diagnosis is made using both clinical and serological features. Renal involvement—manifesting as renal failure or urine dipstick abnormalities—may be the presenting feature of CTD. Renal and general physicians need to have a good understanding of how serological testing can help in the diagnosis of CTD.

Here we consider how anti-nuclear antibody (ANA) and extractable nuclear antigen (ENA) testing can aid the diagnosis of CTD in patients with renal disease. We review how these assays are performed and the principles of serological testing. We summarize the clinical features, renal pathology and diagnostic performance of ANA and ENA testing in a range of CTDs. (We do not discuss lupus nephritis, which is comprehensively covered elsewhere in the literature.)

### How are ANA and ENA assays performed?

ANAs are directed towards components of the cell nucleus. They are detected by indirect immunofluorescence using the human epidermoid carcinoma (HEp-2) cell line. HEp-2 cells, with large nuclei containing many autoantigens, are well suited for the detection of autoantibodies. Patient serum is incubated with the cells before a fluorescent antibody to human immunoglobulin is added. Samples are tested first at a screening dilution (e.g. 1:80) and, if positive, are sequentially diluted until fewer than half the cells are positively stained. This dilution is reported to reflect the strength of ANA binding present in the serum.

Weakly positive ANA titres are common and are often not clinically significant [1]. In addition to the titre, most laboratories report the staining pattern of the antibody, for example, homogeneous, speckled, centromere or nucleolar. This reflects different nuclear proteins to which the antibody has bound (Table 1).

**Table 1:** Prevalence and associations of antinuclear autoantibodies in CTDs

CTD	Autoantibody	Prevalence in disease (%)	Sensitivity (%)	Specificity (%)	Staining pattern	Antibody association
Sjögren’s syndrome	Anti-SSA Anti-SSB	50–70 25–40	8–70 16–40	87 94	Speckled nuclear Speckled nuclear	Neonatal heart block
Limited cutaneous scleroderma	Anti-centromere Anti-Th/To Anti-U1RNP	30 5 5	31	97	Centromere Nucleolar Speckled nuclear	Pulmonary hypertension, peripheral vascular disease Pulmonary fibrosis Mixed connective tissue disorder
Diffuse cutaneous scleroderma	Anti-topoisomerase 1 (anti-Scl-70) Anti-RNA polymerase III Anti-U3RNP (fibrillarin)	30 15 5	26 12	99.5 96	Various Nucleolar Nucleolar	Pulmonary fibrosis, cardiac involvement Greatest association with scleroderma renal crisis (in 52% of patients) More common in African Americans and males; pulmonary hypertension and skeletal muscle involvement
Mixed connective tissue disorder	Anti-U1RNP	100			Speckled nuclear	Usually required for diagnosis
Polymyositis Dermatomyositis	Anti-Jo-1	20			Cytoplasmic	Polymyositis > dermatomyositis Anti-synthetase syndrome: myositis, idiopathic interstitial lung disease, arthritis and Raynaud’s phenomenon

Specific nuclear antigens are associated with different CTDs. Such antigens were historically removed from the nucleus using salt extraction, giving the name ENAs, but are now commonly measured using a solid-phase enzyme-linked immunosorbent assay technique. These include Sjögren's syndrome–related antigen A (SSA; also known as Ro), Sjögren's syndrome–related antigen B (SSB; also known as La), Smith (Sm), topoisomerase 1, ribonucleic acid (RNA) polymerase III, Jo-1 and U1 ribonucleic protein (U1RNP). The antigen panel used for an ENA screen varies between laboratories. For specific clinical questions or in the case of diagnostic uncertainty, it can be helpful to discuss extended screening with a local immunologist.

### Principles of test interpretation

With any test, it is important to understand the sensitivity and specificity and positive and negative predictive values (PPV and NPV, respectively). Sensitivity and specificity are often generated from case–control studies and, depending on the clinical characteristics and disease prevalence, it is possible to overestimate the PPV [2]. Furthermore, these are not necessarily intuitive concepts, and clinicians often overestimate the chance of disease with a positive test result [3].

The diseases we discuss have a low prevalence and the PPV of ANA is poor if applied to the general population. Testing only adds value in the presence of a reasonably high pre-test probability, and the results need to be carefully interpreted with knowledge of the clinical context.

In the remainder of this article we consider individual CTDs. We summarize the salient clinical features (i.e. features that might prompt autoantibody testing) and then discuss the diagnostic performance of ENA tests in that disease.

### Sjögren’s syndrome

Sjögren’s syndrome is characterized by B-cell activation and lymphocytic infiltration of exocrine glands, causing keratoconjunctivitis sicca and xerostomia. Renal involvement occurs in up to 20% of patients and can predate the clinical diagnosis in 30%. In 75% of cases there is an acute or chronic tubulointerstitial nephritis with a CD4^+^ T-cell infiltrate. There may be also tubular defects, typically distal hypokalaemic renal tubular acidosis. Proximal tubular dysfunction can occur, but rarely as full-blown Fanconi syndrome. The remaining 25% of patients have glomerular disease, most commonly immune complex–mediated membranoproliferative glomerulonephritis (MPGN) [4].

The most common ENAs are anti-SSA (in 50–70% of patients) and anti-SSB (in 25–40%). While it is possible to have anti-SSA but negative anti-SSB, the converse is rare. Their presence correlates to diagnosis at a younger age, longer disease duration and more severe exocrine gland involvement. Anti-SSA immunoglobulin G can cross the placenta and cause neonatal heart block. While anti-SSA can also be found in other CTDs such as systemic lupus erythematosus (SLE), anti-SSB is more specific for Sjögren’s syndrome (Table 1) [5].

### Systemic sclerosis

Systemic sclerosis (SSc) is characterized by an overproduction of extracellular matrix proteins and collagen resulting in tissue fibrosis. Vascular proliferation leads to an obliterative vasculopathy. Features include Raynaud’s phenomenon, pulmonary fibrosis, pulmonary hypertension, oesophageal dysmotility and scleroderma renal crisis. It is classified as diffuse cutaneous SSc (dcSSc; skin involvement proximal to the elbows and knees) or limited cutaneous SSc [lcSSc; distal skin involvement, also known as CREST (calcinosis, Raynaud's phenomenon, esophageal dysmotility, sclerodactyly, and telangiectasia) syndrome].

Scleroderma renal crisis develops in 5–10% of patients with dcSSc. This is defined by new-onset accelerated hypertension and rapidly progressive renal impairment (with microangiopathic haemolytic anaemia in 50% of patients). Renal biopsy shows a thrombotic microangiopathy with mucinoid hyperplasia and fibrinoid necrosis that progresses to a proliferative arteriolopathy with characteristic onion-skin appearance in arcuate and interlobular arteries.

Patients with SSc may develop other renal lesions, including penicillamine-related membranous nephropathy, scleroderma-associated vasculopathy and myeloperoxidase-positive vasculitis [6].

The predominant ENAs are anti-centromere and anti-Th/To in lcSSc and anti-topoisomerase 1 and anti-RNA polymerase III in dcSSc. Anti-U1RNP, U3RNP and PM-Scl are found in SSc overlap syndromes. Anti-centromere is specific (97%) for SSc when tested in patients with other CTDs and has a PPV of 89.5%. Their presence is associated with peripheral arterial disease and pulmonary arterial hypertension. Anti-Th/To has been less widely studied and the sensitivity and specificity are not accurately known, but they are present in ∼5% of patients and associated with pulmonary hypertension and fibrosis [7]. Anti-topoisomerase 1 is highly specific (99.5%), with an excellent PPV of 98% but a lower sensitivity (26%). It is associated with cardiac involvement and pulmonary fibrosis. Anti-RNA polymerase III has the strongest association with scleroderma renal crisis, found in ∼50% of patients developing this complication [8].

### Polymyositis and dermatomyositis

These are inflammatory disorders of skeletal muscle, presenting with symmetrical proximal muscle weakness. Dermatomyositis occurs as a result of a complement-mediated microangiopathy and can be associated with underlying malignancy or an overlap with mixed connective tissue disorder. Polymyositis is characterized by an inflammatory infiltrate of CD8^+^ T cells in muscle fibres [9]. Renal manifestations are most commonly acute kidney injury secondary to rhabdomyolysis and myoglobinuria. Chronic glomerulonephritis can occur. MPGN is most commonly described in polymyositis and membranous nephropathy in dermatomyositis [10].

Anti-Jo-1 is an antibody against histidyl-tRNA synthetase and is found in ∼20% of patients [9]. It can also be found in patients with interstitial lung disease without myositis.

### Mixed connective tissue disorder

Mixed connective tissue disorder has overlapping features of SLE, SSc and dermatomyositis. Anti-U1RNP is suggestive of the diagnosis. Renal involvement occurs less frequently than in other disorders. The most common lesion is membranous nephropathy [7]. As mixed CTD can evolve into SLE or SSc, patients can develop lupus nephritis or scleroderma-related nephropathy.

## CONCLUSIONS

Renal involvement is common in CTDs and causes a range of glomerular, tubulointerstitial and vascular pathologies. Autoantibody testing can play an important diagnostic role in patients with multisystem symptoms with renal impairment or dipstick abnormalities. However, their effective clinical utility depends on a reasonably high working pre-test probability of disease, as serological tests are not generally well-performing screening tools in wider populations.

Close liaison with the immunology laboratory can be helpful in evaluating patients whose clinical picture and serological test results are discordant. In some cases, testing for extended autoantibody panels is helpful in reaching a firmer diagnosis. In other cases, alternative diagnoses can lead to unexpected serological test results.

We summarize the diagnostic performance of ANA and ENA tests in Table 1.

## FUNDING

RWH is funded by a Clinical Research Career Development Fellowship from the Wellcome Trust (award number 209562/Z/17/Z).

## CONFLICT OF INTEREST STATEMENT

None declared. The results presented in this article have not been published previously in whole or part, including in abstract format.
